# Supplementary material for: Association of immunoglobulin G N-glycosylation with carotid atherosclerotic plaque phenotypes and actual clinical cardiovascular events: a study protocol for a longitudinal prospective cohort study
Source: BMJ Open. 2022 Jul 22;12(7):e058922. doi: 10.1136/bmjopen-2021-058922 (PMC9316026; doi:10.1136/bmjopen-2021-058922)
Supplement: Author's manuscript [file bmjopen-2021-058922.draft_revisions.pdf]

BMJ Open

BMJ Open is committed to open peer review. As part of this commitment we make the peer review history of every article we publish publicly available.

When an article is published we post the peer reviewers' comments and the authors' responses online. We also post the versions of the paper that were used during peer review. These are the versions that the peer review comments apply to.

The versions of the paper that follow are the versions that were submitted during the peer review process. They are not the versions of record or the final published versions. They should not be cited or distributed as the published version of this manuscript.

BMJ Open is an open access journal and the full, final, typeset and author-corrected version of record of the manuscript is available on our site with no access controls, subscription charges or pay-per-view fees (http://bmjopen.bmj.com).

If you have any questions on BMJ Open's open peer review process please email info.bmjopen@bmj.com

BMJ Open

Association of immunoglobulin G N-glycosylation with carotid atherosclerotic plaque phenotypes and actual clinical cardiovascular events: a study protocol for a longitudinal prospective cohort study

Journal:	*BMJ Open*
Manuscript ID	bmjopen-2021-058922
Article Type:	Protocol
Date Submitted by the Author:	04-Nov-2021
Complete List of Authors:	Tian, Cuihong; The First Affiliated Hospital of Shantou University Medical College, Department of Cardiovascular Medicine<br>Mahara, Gehendra ; The First Affiliated Hospital of Shantou University Medical College<br>Zhang, Hongxia; The First Affiliated Hospital of Shantou University Medical College, Healthcare Center<br>Tan, Xuerui; The First Affiliated Hospital of Shantou University Medical College, Department of Cardiovascular Medicine; The First Affiliated Hospital of Shantou University Medical College, Clinical Research Center
Keywords:	Cardiology < INTERNAL MEDICINE, Protocols & guidelines < HEALTH SERVICES ADMINISTRATION & MANAGEMENT, CARDIOLOGY

SCHOLARONE™
Manuscripts

Association of immunoglobulin G N-glycosylation with carotid atherosclerotic plaque phenotypes and actual clinical cardiovascular events: a study protocol for a longitudinal prospective cohort study

Cuihong Tian[1], Gehendra Mahara[2], Hongxia Zhang[3], Xuerui Tan[1,2*]

[1]Department of Cardiovascular Medicine, The First Affiliated Hospital of Shantou University Medical College, Shantou 515041, Guangdong, China;

[2]Clinical Research Center, The First Affiliated Hospital of Shantou University Medical College, Shantou 515041, Guangdong, China;

[3]Healthcare Center, The First Affiliated Hospital of Shantou University Medical College, Shantou 515041, Guangdong, China.

***Correspondence to**

Prof Xuerui Tan, Clinical Research Center, The First Affiliated Hospital of Shantou University Medical College, Shantou 515041, Guangdong, China; xrtan1@stu.edu.cn

For peer review only

ABSTRACT

Introduction Immune inflammatory response has been shown to play a key role in the pathogenesis of atherosclerosis. Immunoglobulin G (IgG) N-glycosylation has been reported to associate with a 10-year atherosclerotic cardiovascular disease (ASCVD) risk score and subclinical atherosclerosis. However, the relationship of IgG

glycosylation with actual clinical cardiovascular disease (CVD) events and plaque phenotypes has rarely been investigated. Therefore, this study aims to understand whether IgG glycosylation traits are correlated with actual clinical CVD events and plaque phenotypes.

Methods and analysis Designed to verify the efficacy of IgG glycosylation as a risk for CVD events and screen potential biomarkers of CVD to prevent atherosclerosis (AS) occurrence, this longitudinal prospective cohort study will be conducted at The First Affiliated Hospital of Shantou University Medical College, China, with 392 participants having carotid atherosclerosis (CAS) and 392 participants without CAS. The CAS groups will be divided into different subgroups according to the characteristics of plaque phenotype. The ultra-performance liquid chromatography (UPLC) will be used to separate and detect the serum IgG N-glycans. The primary outcome is the actual clinical CVD composite events, consisting of nonfatal myocardial infarction, coronary heart disease death, and fatal or nonfatal stroke in three years.

Ethics and dissemination The Clinical Research Ethics Committee of The First Affiliated Hospital of Shantou University Medical College has approved the ethical approval for this study (Number: B-2021-127). Findings of this study will be submitted for publication in peer-reviewed journals.

Trial Registration number ChiCTR2100048740.

Keywords Immunoglobulin G, Glycosylation, Carotid atherosclerosis, Cardiovascular disease

Word count 2623 words.

For peer review only

Article Summary

Strengths and limitations of this study

- This project is combines a case-control study and prospective cohort study to understand whether IgG N-glycosylation traits correlate with actual clinical CVD events and plaque phenotypes.
- This project is a longitudinal study with three years of follow-up, aiming to investigate the changing trend of IgG N-glycans in people with CAS.
- This research with a novel perspective might provide theoretical evidence for screening the drug targets for pharmaceutical therapy and further understanding the pathogenesis of AS.
- This cohort is derived from a single center and represents only one ethnic group Han Chinese, which may lack ethnic diversity and not apply to the general population.
- Some biases such as loss of follow-up and other uncontrollable factors may exist, although the trial is designed to control the biases.

INTRODUCTION

Background and scope

Atherosclerosis (AS) has a high morbidity and is the leading cause of mortality of cardiovascular disease (CVD), accounting for approximately 20% of all deaths worldwide [1]. Ischemic heart disease (IHD), ischemic stroke, and peripheral vascular

For peer review only

disease (PVD) are the main serious outcomes caused by AS [2]. However, the pathogenesis of AS has not been fully understood so far. The main risk factors for AS are family history, hypertension, smoking, dyslipidemia, diabetes, obesity. Other factors, such as metabolism disorders, platelet activation, thrombosis, intimal damage, inflammatory response, oxidative stress, and vascular smooth muscle cell (VSMC) activation jointly promote the occurrence and progression of AS [3 4]. In addition, whether some novel factors, such as suboptimal health status (SHS) and telomere length in peripheral blood leukocytes could increase the risk of chronic diseases like stroke is being investigated [5 6].

Studies have shown that inflammation is involved in the whole process of the occurrence and development of AS, and specifically, immune inflammation exerts a significant role in this process [7]. Animal experiments have shown that the initiation, growth, differentiation, and rupture of atherosclerotic plaques are influenced by the immune system [8]. A randomized controlled trial (RCT) involving 10,061 patients demonstrated that targeted immune anti-inflammatory pathway therapy effectively reduces CVD events, independent of lowering lipid levels [9]. Furthermore, germinal center-derived immunoglobulin G (IgG) is a determinant factor of plaque size and stability of AS and significantly affects maintaining the molecular properties of the aorta [10]. Therefore, the interaction of the adaptive immune system modulates the progression of AS.

For peer review only

Glycosylation is the most abundant and diverse form of post-transcriptional modification (PTM) in organisms involved in almost every physiological process, including immune responses [11 12]. Alterations in protein glycosylation are considered the major event in the transition from healthy to disease status [12]. Driven by specific enzymes, the complex carbohydrates attaching immunoglobulins have specific regulatory effects, which lead to differences in immune function [13]. A study has shown that glycosylation changes, especially for the most abundant IgG in circulation, directly affect its inflammatory properties [14]. For example, the anti-inflammatory and pro-inflammatory activity of IgG are determined by sialylation and degalactosylation, respectively [15 16]. Furthermore, a cross-sectional study indicates that the deficiency of galactose and sialic acid correlate with blood lipids levels [17]. Therefore, considering the significant role of inflammation, especially immune inflammation in CVD development, different IgG glycosylation traits may stratify the risk phenotypes for CVD development.

Phenotypes of AS plaque include plaque with or without, plaque stability, size, location, and number. A cross-sectional study based on two independent cohorts reported that the glycosylation traits of IgG are associated with the 10-year atherosclerotic cardiovascular disease (ASCVD) risk score, subclinical AS, and the risk factors of CVD, such as smoking, high-density lipoprotein cholesterol (HDL-c), and type 2 diabetes mellitus (T2DM) [18]. However, it is unclear whether IgG glycosylation correlates with actual clinical CVD events and plaque phenotypes. Thus, we hypothesize that IgG N-

For peer review only

glycan profiling is an independent risk factor for actual clinical CVD complicated events.

Objectives

We aim to understand whether IgG glycosylation traits are correlated with plaque phenotypes and actual clinical CVD complicated events, including nonfatal myocardial infarction (MI), coronary heart disease (CHD) death, and fatal or nonfatal stroke in three years.

METHODS AND ANALYSIS

Design and setting

This study is an observational, single-center longitudinal prospective cohort trial conducted at The First Affiliated Hospital of Shantou University Medical College (SUMC), Guangdong, China. The protocol applies the checklists of SPIRIT 2013 Statement [19], a guideline of clinical trials' writing contents.

Study participants

This study will be conducted in the Healthcare Center of The First Affiliated Hospital of SUMC. After the clinical study registered on July 15, 2021, participants undergoing carotid artery ultrasound examination will be divided into CAS group and control group according to recruitment protocol (Figure 1). Potential eligible participants will be recorded in real-time by a sonographer. The research team consists of physicians

For peer review only

familiar with ultrasound examination and skilled academic staff in clinical study design and statistical analysis. Furthermore, the investigators will get the required research-related training before the study start.

Inclusion criteria

Prospective participants will be carefully evaluated based on the following inclusion criteria:

1. Age between 50 to 65 years old;
2. Carotid ultrasound is performed;
3. Participants or their families provide informed consent.

Exclusion criteria

Participants with any of the following cannot be enrolled in this study:

1. Participants complicated with IHD, stroke, infection, immune system disease, blood system disease, tumor;
2. History of lipid-lowering therapy;
3. Irregular follow-up.

Withdrawal

1. Participants may withdraw their participation from the study for any reason; and
2. Other circumstances: based on the investigator's judgment, the participant no longer meets the study criteria for any reason.

For peer review only

Informed consent

Before enrollment, the investigator will introduce the main purpose and content of the study to the participants. In addition, participants will be informed of the possible risks and benefits of the research and ensure that their participation is completely voluntary. After signing the informed consent, all study-related including enrolled participants' personal information, will be kept confidential. This study will be carried out in accordance with the principles of the Declaration of Helsinki.

Blinding

Trial participants, sonographers, data gatherers, and data analysts are not blinded to group assignments, while specimen examiners and follow-up staffers are blinded.

Data collection and management

The information of the enrolled participants, including smoking history, diabetes status, antihypertensive medication, low-density lipoprotein (LDL), HDL, triacylglycerol (TG), and total cholesterol (TC) level will be assembled. In addition, a sonographer will inspect the characteristics of carotid plaque, which consists of plaque size, number, situation, blood flow, echo, shape, and stability. All data will be kept through Epidata (Denmark, version 3.1), an Electronic Data Capture (EDC), applied for data entry. Furthermore, 1ml serum will be collected from each participant at different times (enrollment, 1st year, 2nd year, and 3rd year) after being centrifuged with 3000r/min of rotate speed lasting for 10 minutes and stored in the freezer at -80℃ for biochemistry

For peer review only

and glycan analysis.

Follow-up

The survival status (surviving/deceased) and actual clinical CVD complicated events of the participants with CAS, including CVD death, MI, and stroke, will be followed up at the end of the first, second, and third years by two trained investigators through telephone, outpatient visit, and physical examination. Furthermore, the serum samples will be collected every year, used for the detection of (Figure. 2).

Sample size

It has been reported that the incidence of CVD events in the healthy control group and the CAS group is 4.0% and 9.5%, respectively [20]. Set as α=0.05 on a two-sided test and 80% power to obtain a significant statistic difference, 652 participants will be needed with 326 for each group. Growth of one-fifth is required for the total sample size when considering the cases of lost follow-up and refusal to visit. Finally, a total of 784 participants of the total sample size is required, contributing to at least 392 per arm of the study. The sample size is calculated using PASS software (NCSS, America, version 11).

Carotid ultrasound

Color Doppler ultrasonography (Siemens Acuson X150) will be used to scan the carotid artery. Participants will be held on a supine position with the head turned to the opposite

For peer review only

side and the probe from the root of the neck to the head. The common carotid artery, bifurcation, internal carotid artery, and external carotid artery are scanned, respectively. Based on the diameter, plaque morphology, and echo characteristics, these plaques are classified into three types (soft plaques: uniform or uneven images, irregular form, weak and low echo; Hard spots: strong echo, uneven thickening of the tube wall with enhanced echo and sound shadow behind; Mixed spot: uneven echo, unequal intensity, irregular shape, large image range). Hard plaques are defined as stable plaques, while soft and mixed plaques are regarded as unstable plaques.

Ultrahigh Pressure Liquid Chromatograph

IgG glycosylation will be detected by Ultrahigh Pressure Liquid Chromatograph (UPLC) (Shimazu, LC-30AD) technique. Firstly, IgG will be isolated with the Protein G extraction plate. After being dried and exposed to peptide-N-glycosidase F (PNGcase F), N-glycan will be released and labeled with 2-aminobenzamide (2-AB) fluorescent dye. Furthermore, the free markers and reducing agents will be removed by hydrophilic chromatography-solid phase extraction. After that, fluorescent-labeled N-glycans will be separated by hydrophilic chromatography on UPLC [21]. Finally, the automatic processing method with a traditional integration algorithm is used for data processing, and each chromatogram is manually corrected to ensure the same integration interval for all samples.

Outcome measurements

For peer review only

The primary outcome of this study is the composite events of CVD in three years. The secondary outcome is the 3-year mortality by counting all-cause of death.

Statistical and analytical plans

The categorical variables will be expressed by frequency (percentage), and univariate analysis will be performed by chi-square test. The data of normal distribution are shown as mean ± standard deviation. T-test and analysis of variance are used for comparison of two groups and multiple groups, respectively. Non-normal distribution data are represented by median (P25, P75). Univariate analysis is performed by non-parametric test. Interpolation is carried out according to the type of data loss. If the lost data is of numerical type, the average value is used to fill it. However, if the lost data is non-numerical, the value with the highest occurrence frequency in this index is used. Logistic regression will be used to analyze the association between IgG N-glycosylation characteristics and CVD events. Subsequently, a glycan risk score will be created to evaluate the associative effects of all glycan traits screened. P-value <0.05 indicates statistical significance. SPSS software version 20.0 will be used for statistical analysis.

Monitoring

This research will be conducted critically according to the approved proposal. The data and safety monitoring committee (DMC) of our hospital will undertake the responsibility of data review and interpretation, which is beneficial to the security and

For peer review only

privacy of participants and the validity and integrity of data. Six researchers, independent of the sponsor and conflict of interest, make up the DMC, which will evaluate the data after a quarter, a half, and three-quarters of the participants enrolled in this study voluntarily. The six researchers will be involved in the whole research progress. Furthermore, we will launch the audit trails to trace the change and guarantee the quality of research data.

Auditing

Based on state regulations, the audit works will be conducted during the entire study progress. Representatives in the ethics committee will shoulder the obligation to inform the related parties of all proposal revisions.

Harms

This non-intervention trial is remarkably safe. Participants will get no exposure to any drug-related or surgical-related events.

Amendment

Any modifications of the proposal will be submitted to Clinical Research Ethics Committee and DMC for consent.

Discussion

AS leads to acute myocardial infarction (AMI), cerebral infarction, and other serious

For peer review only

consequences, which seriously affect life quality and bring a huge economic burden to patients and society. It was reported that at the beginning of the 2010s, the total hospitalization costs of patients hospitalized for CVDs were over 40 billion Yuan, which contributes to more than 1.60% of national health expenditures [22]. Therefore, ASCVD has become a major public health problem in China and worldwide, and the prevention and treatment of CVD are urgent. Although the mechanism of AS is currently unknown, numerous evidence show that the pathogenesis of AS is mainly related to the adaptive immune-inflammatory response. The joint action between immune cells is the key link in the occurrence and development of AS [23]. Therefore, the control of adaptive immune response through immune regulatory strategies or vaccination is a potential therapeutic direction for AS.

AS contains IgG antibodies against LDL, oxidized LDL (ox-LDL), and apolipoprotein B (ApoB) [24]. Some studies suggested that IgG antibody titer is positively correlated with AS whether in mice or humans [25], and anti-ApoB IgG antibody can aggravate AS [26]. Furthermore, the removal of glycans contributes to the disappearance of the anti-inflammation of IgG [27]. While the enrichment of sialic acid is responsible for a tenfold growth of anti-inflammation of IgG [28]. However, the role of IgG and its glycosylation in AS remains unclear. Therefore, clarifying the correlation between IgG and AS, and revealing the mechanism of its occurrence and development of AS in humans are essential to provide a new direction for the treatment of AS and prevent further complications. It has important scientific significance and social and economic value

For peer review only

for improving the quality of life, decreasing the mortality rate, and reducing the social and economic burden of CVD patients.

This trial aims to assess the association between IgG N-glycosylation and CVD events of AS patients. If the results of this study show a significant correlation between IgG glycosylated traits and CVD events, it will provide further evidence on the role of glycosylation modification and immune-inflammatory response in AS.

TRIAL STATUS

Participants recruitment began on 15 July 2021 and will be finished on 31 December 2021.

Acknowledgments We are grateful to Xingyu Wang for his helpful guidance on the revision of the protocol.

Author Contributions CT was responsible for the design and draft of the manuscript. GM and HZ contributed to the revision of the manuscript. XT acted as principal investigator and helped to conceive and revise the protocol. The final version of the manuscript was critically reviewed and approved by all authors.

Funding This work is supported by the National Nature Science Foundation of China (Grant no: 82073659) and the Science and Technology Special Fund of Guangdong Province in 2021.

For peer review only

Competing interests The authors declare that they have no competing interests.

Participants and public involvement Participants and/or the public were not involved in the design, or conduct, or reporting, or dissemination plan of this research.

Participants consent for publication Not required.

Ethics approval The study protocol has been approved by the Committee of Ethics of The First Affiliated Hospital of SUMC (Number: B-2021-127). Informed consent will be obtained from all participants.

Provenance and peer review Not commissioned; externally peer-reviewed.

Open access This is an open access article distributed in accordance with the Creative Commons Attribution Non-Commercial (CC BY- NC 4.0) license, which permits others to distribute, remix, adapt, build upon this work non-commercially, and license their derivative works on different terms, provided the original work is properly cited, appropriate credit is given, any changes made indicated, and the use is non-commercial. See: http:// creativecommons. org/ licenses/ by- nc/ 4. 0/.

Consent for publication All authors and investigators gave their consent to publication.

Availability of data and materials The project owner has access to the final trial dataset. The trial

For peer review only

data will be made public in the EDC system, where participants' names, phone numbers, and other personal information will be removed to protect their privacy. Moreover, manuscript(s) will be published in a peer review clinical journal regardless of whether the trial results are positive, negative, or inconclusive. With a reasonable request, the public is available to these data under the permission of the authors or correspondence author.

REFERENCES

1. Wang Y, Xie Y, Zhang A, et al. Exosomes: An emerging factor in atherosclerosis. *Biomed Pharmacother* 2019;115:108951. doi: 10.1016/j.biopha.2019.108951 [published Online First: 2019/05/12]
2. Libby P, Buring JE, Badimon L, et al. Atherosclerosis. *Nat Rev Dis Primers* 2019;5(1):56. doi: 10.1038/s41572-019-0106-z [published Online First: 2019/08/20]
3. Gupta M, Blumenthal C, Chatterjee S, et al. Novel emerging therapies in atherosclerosis targeting lipid metabolism. *Expert Opin Investig Drugs* 2020;29(6):611-22. doi: 10.1080/13543784.2020.1764937 [published Online First: 2020/05/05]
4. Kattoor AJ, Pothineni NVK, Palagiri D, et al. Oxidative Stress in Atherosclerosis. *Current atherosclerosis reports* 2017;19(11):42. doi: 10.1007/s11883-017-0678-6 [published Online First: 2017/09/19]
5. Wang Y, Ge S, Yan Y, et al. China suboptimal health cohort study: rationale, design and baseline characteristics. *J Transl Med* 2016;14(1):291. doi: 10.1186/s12967-016-1046-y [published Online First: 2016/10/16]
6. Cao W, Zheng D, Zhang J, et al. Association between telomere length in peripheral blood leukocytes and risk of ischemic stroke in a Han Chinese population: a linear and non-linear Mendelian randomization analysis. *J Transl Med* 2020;18(1):385. doi: 10.1186/s12967-020-02551-1 [published Online First: 2020/10/14]
7. Sage AP, Mallat Z. Readapting the adaptive immune response - therapeutic strategies for atherosclerosis. *Br J Pharmacol* 2017;174(22):3926-39. doi: 10.1111/bph.13700 [published Online First: 2017/01/05]
8. Libby P, Lichtman AH, Hansson GK. Immune effector mechanisms implicated in atherosclerosis: from mice to humans. *Immunity* 2013;38(6):1092-104. doi: 10.1016/j.immuni.2013.06.009 [published Online First: 2013/07/03]
9. Ridker PM, Everett BM, Thuren T, et al. Antiinflammatory Therapy with Canakinumab for Atherosclerotic Disease. *The New England journal of medicine* 2017;377(12):1119-31. doi: 10.1056/NEJMoa1707914 [published Online First: 2017/08/29]
10. Centa M, Jin H, Hofste L, et al. Germinal Center-Derived Antibodies Promote Atherosclerosis Plaque Size and Stability. *Circulation* 2019;139(21):2466-82. doi: 10.1161/circulationaha.118.038534 [published Online First: 2019/03/22]

For peer review only

11. Molinari M. N-glycan structure dictates extension of protein folding or onset of disposal. *Nat Chem Biol* 2007;3(6):313-20. doi: 10.1038/nchembio880 [published Online First: 2007/05/19]

12. Ohtsubo K, Marth JD. Glycosylation in cellular mechanisms of health and disease. *Cell* 2006;126(5):855-67. doi: 10.1016/j.cell.2006.08.019 [published Online First: 2006/09/09]

13. Maverakis E, Kim K, Shimoda M, et al. Glycans in the immune system and The Altered Glycan Theory of Autoimmunity: a critical review. *J Autoimmun* 2015;57:1-13. doi: 10.1016/j.jaut.2014.12.002 [published Online First: 2015/01/13]

14. Chan AC, Carter PJ. Therapeutic antibodies for autoimmunity and inflammation. *Nat Rev Immunol* 2010;10(5):301-16. doi: 10.1038/nri2761 [published Online First: 2010/04/24]

15. Washburn N, Schwab I, Ortiz D, et al. Controlled tetra-Fc sialylation of IVIg results in a drug candidate with consistent enhanced anti-inflammatory activity. *Proc Natl Acad Sci U S A* 2015;112(11):E1297-306. doi: 10.1073/pnas.1422481112 [published Online First: 2015/03/04]

16. Nimmerjahn F, Anthony RM, Ravetch JV. Agalactosylated IgG antibodies depend on cellular Fc receptors for in vivo activity. *Proc Natl Acad Sci U S A* 2007;104(20):8433-7. doi: 10.1073/pnas.0702936104 [published Online First: 2007/05/09]

17. Liu D, Chu X, Wang H, et al. The changes of immunoglobulin G N-glycosylation in blood lipids and dyslipidaemia. *J Transl Med* 2018;16(1):235. doi: 10.1186/s12967-018-1616-2 [published Online First: 2018/08/31]

18. Menni C, Gudelj I, Macdonald-Dunlop E, et al. Glycosylation Profile of Immunoglobulin G Is Cross-Sectionally Associated With Cardiovascular Disease Risk Score and Subclinical Atherosclerosis in Two Independent Cohorts. *Circulation research* 2018;122(11):1555-64. doi: 10.1161/circresaha.117.312174 [published Online First: 2018/03/15]

19. Chan AW, Tetzlaff JM, Altman DG, et al. SPIRIT 2013 statement: defining standard protocol items for clinical trials. *Ann Intern Med* 2013;158(3):200-7. doi: 10.7326/0003-4819-158-3-201302050-00583 [published Online First: 2013/01/09]

20. Kotruchin P, Hoshide S, Kario K. Carotid atherosclerosis and the association between nocturnal blood pressure dipping and cardiovascular events. *J Clin Hypertens (Greenwich)* 2018;20(3):450-55. doi: 10.1111/jch.13218 [published Online First: 2018/02/17]

21. Liu D, Xu X, Li Y, et al. Immunoglobulin G N-Glycan Analysis by Ultra-Performance Liquid Chromatography. *J Vis Exp* 2020(155) doi: 10.3791/60104 [published Online First: 2020/02/06]

22. Hu SS, Kong LZ, Gao RL, et al. Outline of the report on cardiovascular disease in China, 2010. *Biomed Environ Sci* 2012;25(3):251-6. doi: 10.3967/0895-3988.2012.03.001 [published Online First: 2012/07/31]

23. Wolf D, Ley K. Immunity and Inflammation in Atherosclerosis. *Circulation research* 2019;124(2):315-27. doi: 10.1161/circresaha.118.313591 [published Online First: 2019/01/18]

24. Kimura T, Tse K, Sette A, et al. Vaccination to modulate atherosclerosis. *Autoimmunity* 2015;48(3):152-60. doi: 10.3109/08916934.2014.1003641 [published Online First: 2015/02/17]

25. Björkbacka H, Alm R, Persson M, et al. Low Levels of Apolipoprotein B-100 Autoantibodies Are Associated With Increased Risk of Coronary Events. *Arteriosclerosis, thrombosis, and vascular biology* 2016;36(4):765-71. doi: 10.1161/atvbaha.115.306938 [published Online First:

For peer review only

2016/02/27]
26. Tay C, Liu YH, Kanellakis P, et al. Follicular B Cells Promote Atherosclerosis via T Cell-Mediated Differentiation Into Plasma Cells and Secreting Pathogenic Immunoglobulin G. *Arteriosclerosis, thrombosis, and vascular biology* 2018;38(5):e71-e84. doi: 10.1161/atvbaha.117.310678 [published Online First: 2018/03/31]
27. Kaneko Y, Nimmerjahn F, Ravetch JV. Anti-inflammatory activity of immunoglobulin G resulting from Fc sialylation. *Science (New York, NY)* 2006;313(5787):670-3. doi: 10.1126/science.1129594 [published Online First: 2006/08/05]
28. Schwab I, Nimmerjahn F. Intravenous immunoglobulin therapy: how does IgG modulate the immune system? *Nat Rev Immunol* 2013;13(3):176-89. doi: 10.1038/nri3401 [published Online First: 2013/02/16]

Figure 1. Participants distribution in this study.

Figure 2. Schedule of enrollment, distribution and assessments for the trial.

For peer review only

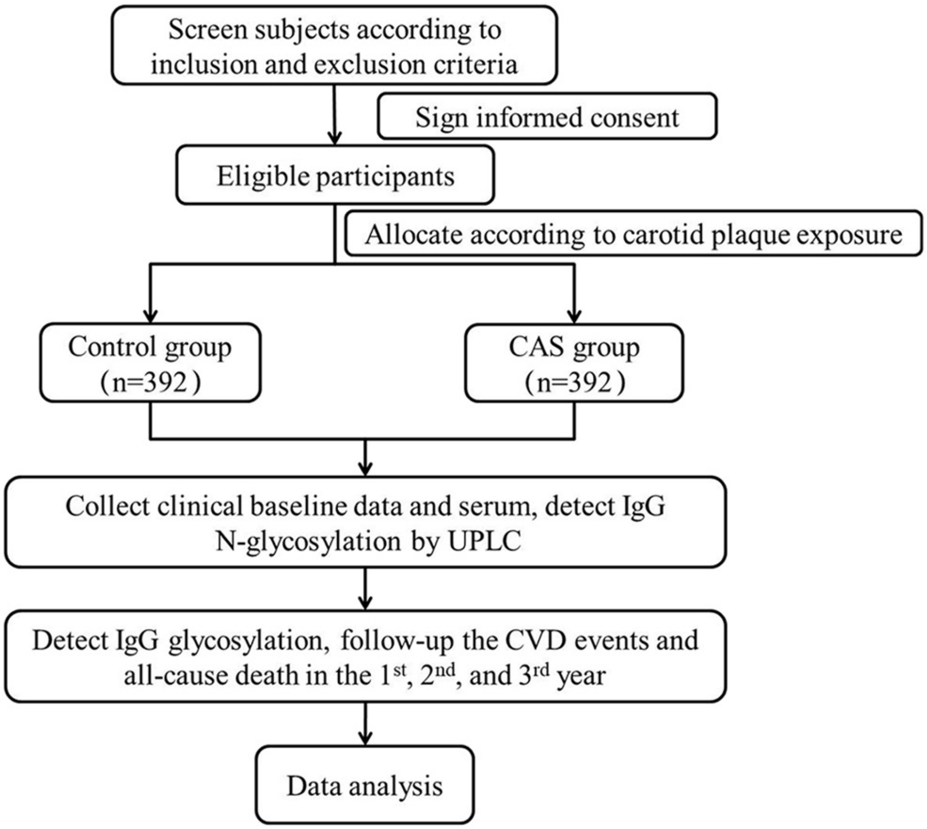

Figure 1. Participants distribution in this study.

90x90mm (300 x 300 DPI)

Study period							
	Enrollment	Distribution	UPLC	Follow-up			
Timepoint	D0	D1	W1	Y1	Y2	Y3	Close-out
Enrollment							
Prescreening	X						
Eligibility screen	X						
Informed consent	X						
Distribution							
Control group		X					
CAS group		X					
Assessments							
Gender		X					
Age		X					
Smoking		X					
Diabetes		X					
Hypotensor		X					
LDL		X					
HDL		X					
TC		X					
TG		X					
Carotid ultrasound							
Size		X					
Number		X					
Situation		X					
Blood flow		X					
Echo		X					
Shape		X					
Stability		X					
Serum		X					
UPLC							
IgG glycosylation			X	X	X	X	
Outcome							
Nonfatal MI				X	X	X	
CHD death				X	X	X	
Stroke				X	X	X	
All-cause death				X	X	X	
Analysis							X

Figure 2. Schedule of enrollment, distribution and assessments for the study.

90x90mm (300 x 300 DPI)

BMJ Open

Association of immunoglobulin G N-glycosylation with carotid atherosclerotic plaque phenotypes and actual clinical cardiovascular events: a study protocol for a longitudinal prospective cohort study

Journal:	*BMJ Open*
Manuscript ID	bmjopen-2021-058922.R1
Article Type:	Protocol
Date Submitted by the Author:	04-May-2022
Complete List of Authors:	Tian, Cuihong; The First Affiliated Hospital of Shantou University Medical College, Department of Cardiovascular Medicine<br>Mahara, Gehendra ; The First Affiliated Hospital of Shantou University Medical College, Clinical Research Center<br>Zhang, Hongxia; The First Affiliated Hospital of Shantou University Medical College, Health Care Center<br>tan, xuerui; The First Affiliated Hospital of Shantou University Medical College, Department of Cardiovascular Medicine; The First Affiliated Hospital of Shantou University Medical College, Clinical Research Center
**Primary Subject Heading**:	Cardiovascular medicine
Secondary Subject Heading:	Cardiovascular medicine, Immunology (including allergy)
Keywords:	Cardiology < INTERNAL MEDICINE, Protocols & guidelines < HEALTH SERVICES ADMINISTRATION & MANAGEMENT, CARDIOLOGY

SCHOLARONE™
Manuscripts

Association of immunoglobulin G N-glycosylation with carotid atherosclerotic plaque phenotypes and actual clinical cardiovascular events: a study protocol for a longitudinal prospective cohort study

Cuihong Tian[1], Gehendra Mahara[2], Hongxia Zhang[3], Xuerui Tan[1,2*]

[1]Department of Cardiovascular Medicine, The First Affiliated Hospital of Shantou University Medical College, Shantou, Guangdong 515041, China;

[2]Clinical Research Center, The First Affiliated Hospital of Shantou University Medical College, Shantou, Guangdong 515041, China;

[3]Health Care Center, The First Affiliated Hospital of Shantou University Medical College, Shantou, Guangdong 515041, China.

***Correspondence to**

Prof. Xuerui Tan, Clinical Research Center, The First Affiliated Hospital of Shantou University Medical College, Shantou, Guangdong 515041, China;

doctortxr@126.com

For peer review only

ABSTRACT

Introduction Immune inflammatory response plays a key role in the pathogenesis of atherosclerosis. Immunoglobulin G (IgG) N-glycosylation is reported to be associated with the 10-year atherosclerotic cardiovascular disease (ASCVD) risk score and

subclinical atherosclerosis. However, the relationship of IgG glycosylation with actual clinical cardiovascular disease (CVD) events and plaque phenotypes has rarely been investigated. Therefore, this study aims to understand whether IgG glycosylation traits are correlated with actual clinical CVD events and plaque phenotypes.

Methods and analysis Designed to verify the efficacy of IgG glycosylation as a risk for CVD events and screen potential biomarkers of CVD to prevent atherosclerosis (AS) occurrence, this longitudinal prospective cohort study will be conducted at the First Affiliated Hospital of Shantou University Medical College, China. In total, 2720 participants routinely examined by carotid ultrasound will be divided into different groups according to plaque phenotype characteristics. Ultra-performance liquid chromatography (UPLC) will be performed to separate and detect IgG N-glycans in serum collected at baseline, and at the end of the 1st, 2nd and 3rd years. The primary outcome is the actual clinical CVD composite events, including nonfatal myocardial infarction, death due to coronary heart disease, and fatal or nonfatal stroke.

Ethics and dissemination The Clinical Ethics Committee of the First Affiliated Hospital of Shantou University Medical College approved this study (Number: B-2021-127). Findings of this study will be submitted for publication in peer-reviewed journals.

Trial Registration number ChiCTR2100048740.

Keywords Immunoglobulin G, Glycosylation, Carotid atherosclerosis, Cardiovascular disease

For peer review only - http://bmjopen.bmj.com/site/about/guidelines.xhtml

Article Summary

Strengths and limitations of this study

- This study is a longitudinal prospective cohort study, which will indicate the change of IgG glycans in people from 50 to 65 years of age.
- The feasibility of this study is high as the study does not require treatment of participants with drugs or surgery.
- Due to the protective effects of estrogen on atherosclerosis, this study will only involve post-menopausal women.
- The cohort is derived from a single center and represents only one ethnic group, Han Chinese, which will lack ethnic diversity and results might not be applicable to the general population.
- Some biases, such as loss of follow-up and other uncontrollable factors, may exist, although this study is designed to control the biases.

INTRODUCTION

Background and scope

Atherosclerosis (AS) is a cardiovascular disease (CVD) with high morbidity and mortality, accounting for approximately 20% of all deaths worldwide[1]. Ischemic heart disease (IHD), ischemic stroke and peripheral vascular disease (PVD) are the main serious outcomes caused by AS[2]. However, the pathogenesis of AS remains obscure. The main risk factors for AS are family history, hypertension, smoking, dyslipidemia, diabetes and obesity. Other factors, such as metabolism disorders, platelet activation,

For peer review only - http://bmjopen.bmj.com/site/about/guidelines.xhtml

thrombosis, intimal damage, inflammatory response, oxidative stress and vascular smooth muscle cell (VSMC) activation jointly promote the occurrence and progression of AS[3 4]. In addition, whether some novel factors, such as suboptimal health status (SHS) and telomere length in peripheral blood leukocytes, could increase the risk of ASCVD is being investigated[5 6].

Studies show that immune inflammation exerts a significant role in atherogenesis[7]. Animal experiments indicate that the initiation, growth, differentiation and rupture of atherosclerotic plaques are influenced by the immune system[8]. A randomized controlled trial (RCT) involving 10,061 patients demonstrated that targeted immune anti-inflammatory pathway therapy significantly reduces CVD events, independent of lowering lipid levels[9]. Furthermore, germinal center-derived immunoglobulin G (IgG) is a determinant of plaque size and stability, and effectively maintains the molecular properties of the aorta[10]. Therefore, the interaction of the adaptive immune system modulates the progression of AS.

Glycosylation is the most abundant and diverse form of post-transcriptional modification in organisms, involved in almost every physiological process, including immune responses[11 12]. Alterations in protein glycosylation are considered as the major event in the transition from health to disease status[12]. It has been demonstrated that IgG glycans are associated with a variety of diseases, such as immune system diseases[13] , cancers[14 15 16], nervous system disease[17]， infectious diseases[18 19], and

For peer review only - http://bmjopen.bmj.com/site/about/guidelines.xhtml

hematological system diseases[20]. Driven by specific enzymes, the complex carbohydrates attached to immunoglobulins have specific regulatory effects, which lead to differences in immune function[21]. A study revealed that glycosylation changes, especially for the most abundant IgG in circulation, directly affect its inflammatory properties[22]. For example, the anti-inflammatory and pro-inflammatory activity of IgG are determined by sialylation and degalactosylation respectively[23 24]. Furthermore, a cross-sectional study indicated that the deficiency of galactose and sialic acid correlates with blood lipid levels[25]. Therefore, considering the significant role of inflammation, especially immune inflammation in CVD development, different IgG glycosylation traits may stratify the risk phenotypes for CVD development.

Phenotypes of plaque include with or without plaque, plaque stability, size, location and number. A cross-sectional study based on two independent cohorts reported that the glycosylation traits of IgG are associated with the 10-year atherosclerotic cardiovascular disease (ASCVD) risk score, subclinical AS and risk factors for CVD, such as smoking, high-density lipoprotein cholesterol (HDL-c) and type 2 diabetes mellitus (T2DM)[26]. However, it is unclear whether IgG glycosylation correlates with actual clinical CVD events and plaque phenotypes. Thus, we hypothesize that IgG N-glycan profiling is an independent prognostic indicator for actual clinical CVD composite events.

For peer review only - http://bmjopen.bmj.com/site/about/guidelines.xhtml

Objectives

We aim to understand whether IgG glycosylation traits are correlated with plaque phenotypes and actual clinical CVD composite events, including nonfatal myocardial infarction (MI), coronary heart disease (CHD) death, and fatal or nonfatal stroke.

METHODS AND ANALYSIS

Design and setting

This study is an observational, single-center longitudinal prospective cohort study to be conducted at the First Affiliated Hospital of Shantou University Medical College (SUMC), Guangdong, China. The protocol applies the checklists of the SPIRIT 2013 Statement[27], a guideline for clinical trial design.

Participants

This study will be conducted in the Health Care Center of the First Affiliated Hospital of SUMC, where people over 50 years old are routinely recommended to be examined by carotid ultrasonography for early diagnosis and detection of CAS, and early prevention of CVD events. After the clinical study registered on July 15, 2021, participants conventionally undergoing carotid artery ultrasound examination will be recruited and divided into different groups according to carotid plaque phenotypes (Figure 1). Potential eligible participants will be recorded in real-time by a sonographer. The research team consists of physicians familiar with ultrasound examination, and academic staff skilled in clinical study design and statistical

For peer review only - http://bmjopen.bmj.com/site/about/guidelines.xhtml

analysis. Furthermore, all investigators will obtain the required research-related training before the start of the study.

Inclusion criteria

Prospective participants will be carefully evaluated based on the following inclusion criteria:

1. Age between 50 to 65 years old;
2. Had carotid ultrasound performed;
3. Participants or their families provided informed consent.

Exclusion criteria

Participants with any of the following will not be enrolled in this study:

1. Participants with complications such as IHD, stroke, infection, immune system disease, nervous system disease, hematological system disease and cancer;
2. History of lipid-lowering therapy;
3. History of anticoagulant therapy or antiplatelet therapy or vasodilator therapy;
4. History of intravenous immunoglobulin (IVIG) or blood transfusion within one month;
5. Irregular follow-up.

Withdrawal

1. Participants may withdraw their participation from the study for any reason; and

For peer review only - http://bmjopen.bmj.com/site/about/guidelines.xhtml

2. Other circumstances: based on the investigator's judgment, the participant no longer meets the study criteria for any reason.

Blinding

Participants, sonographers, data gatherers and data analysts are not blinded to group assignments, whereas specimen examiners and follow-up staffers are blinded.

Data collection and management

Information of the enrolled participants, including smoking history, diabetes status, and antihypertensive medication, along with low-density lipoprotein (LDL), HDL, triacylglycerol (TG) and total cholesterol (TC) levels, will be assembled. In addition, a sonographer will inspect the characteristics of carotid plaque, including plaque size, number, site, blood flow, echo, shape and stability. All data will be kept through EpiData (Denmark, version 3.1), an electronic data capture (EDC), applied for data entry. Furthermore, 1ml serum will be collected from each participant at different times (enrollment, 1st year, 2nd year and 3rd year) after being centrifuged at 604 g for 10 minutes at 277.15K. Serum will be stored in the freezer at -80℃ for biochemistry and glycan analysis.

Follow-up

The survival status (surviving/deceased) and actual clinical CVD composite events of the participants, including CVD death, MI and stroke, will be followed up at the end

For peer review only - http://bmjopen.bmj.com/site/about/guidelines.xhtml

of the first, second and third years by two trained investigators through telephone, outpatient visit and physical examination. Furthermore, serum samples will be collected every year, and used for the detection of IgG glycans (Figure 2).

Sample size

It has been reported that the incidence of CVD events is about 6%[28]. According to Liu et al., among 24 IgG glycans, GP6 is significantly different between people with and without ischemic stroke [5.00(4.26, 5.85) *vs.* 4.48(3.93, 4.35), P=0.028][29], and therefore can be used to roughly estimate the sample size. Converting median and quartile to mean and standard deviation is necessary for the sample size[30-32]. Taking the incidence of CVD events and this conversion into consideration, 2267 participants will be needed, based on an α=0.05 on a two-sided test and 90% power, to obtain a statistical difference. An additional one-fifth is required for the total sample size when considering the cases of lost follow-up and refusal to visit. Finally, a total of 2720 participants are required for the study. The sample size was calculated by using PASS software (NCSS, America, version 11).

Carotid ultrasound

Color Doppler ultrasonography (Siemens Acuson X150) will be used to scan the carotid artery. Participants will be held on a supine position with the head turned to the opposite side and the probe moved from the root of the neck to the head. The common carotid artery, bifurcation, internal carotid artery and external carotid artery

For peer review only - http://bmjopen.bmj.com/site/about/guidelines.xhtml

are scanned. Based on the diameter, plaque morphology and echo characteristics, the plaques are classified into three types (soft plaques: uniform or uneven images, irregular form, weak and low echo; hard plaques: strong echo, uneven thickening of the tube wall with enhanced echo and sound shadow behind; mixed plaques: uneven echo, unequal intensity, irregular shape, large image range). Hard plaques are defined as stable plaques, while soft and mixed plaques are regarded as unstable plaques.

Ultrahigh pressure liquid chromatography

IgG glycosylation will be detected by ultrahigh pressure liquid chromatography (UPLC) (Shimazu, LC-30AD). First, IgG will be isolated with a protein G extraction plate. After being dried and exposed to peptide-N-glycosidase F (PNGase F), N-glycan will be released and labeled with 2-aminobenzamide (2-AB) fluorescent dye. Furthermore, the free markers and reducing agents will be removed by hydrophilic chromatography-solid phase extraction. Subsequently, fluorescently-labeled N-glycans will be separated by hydrophilic chromatography on UPLC [33]. Finally, an automatic processing method with a traditional integration algorithm is used for data processing. Each chromatogram is manually corrected to ensure the same integration interval for all samples.

Outcome measurements

The primary outcome of this study is the composite events of CVD. The secondary outcome is the 3-year mortality by counting all-cause death.

For peer review only - http://bmjopen.bmj.com/site/about/guidelines.xhtml

Statistical and analytical plans

The categorical variables will be expressed by frequency (percentage), and univariate analysis will be performed by the chi-square test. The data of normal distribution are shown as mean ± standard deviation. T-test and analysis of variance are used for comparison of two groups and multiple groups, respectively. Non-normally distributed data are represented by median (P25, P75). Univariate analysis is performed by non-parametric testing. Interpolation is carried out according to the type of data loss. If the lost data is of the numerical type, the average value is used to fill it. However, if the lost data is non-numerical, the value with the highest occurrence frequency in this index is filled in. Logistic regression will be applied to analyze the association between IgG N-glycosylation characteristics and CVD events. Subsequently, a glycan risk score will be created to evaluate the associative effects of all glycan traits screened. A p-value <0.05 indicates statistical significance. SPSS software (version 26.0) will be used for statistical analysis.

Monitoring

This research will be conducted critically according to the approved proposal. The data and safety monitoring committee (DMC) of our hospital will undertake the responsibility of data review and interpretation, which is beneficial to the security and privacy of the participants, and validity and integrity of the data. Six researchers, independent of the sponsor and conflict of interest, make up the DMC, which will

For peer review only

evaluate the data after a quarter, a half and three-quarters of the participants voluntarily enroll in this study. The six researchers will be involved in the entire research progress.

Auditing

Based on state regulations, the audit works will be conducted during the entire study process to trace the changes and guarantee the quality of research data. Representatives on the ethics committee will shoulder the obligation to inform the related parties of all proposal revisions.

Harms

This non-intervention study is remarkably safe. Participants will get no exposure to any drug-related or surgery-related therapies.

Amendment

Any modifications of the proposal will be submitted to the Clinical Ethics Committee and DMC for consent.

ETHICS AND DISSEMINATION

Ethics approval

The study protocol was approved by the Clinical Ethics Committee of the First Affiliated Hospital of SUMC on July 13, 2021 (Number: B-2021-127).

For peer review only - http://bmjopen.bmj.com/site/about/guidelines.xhtml

Informed consent

Before enrollment, the investigator will introduce the main purpose and content of the study to the participants. In addition, participants will be informed of the possible risks and benefits of the research to ensure that their participation is completely voluntary. After participants sign the informed consent, all study-related data, including personal information, will be kept confidential. This study will be carried out in accordance with the principles of the Declaration of Helsinki.

Dissemination

All data and findings of this study will be disseminated at scientific conferences or be submitted for publication in peer-reviewed journals.

DISCUSION

AS can lead to acute myocardial infarction (AMI), cerebral infarction and other serious consequences, which seriously affect quality of life and bring a huge economic burden to patients and society. It has been reported that at the beginning of 2010, the total hospitalization cost of CVD patients was over 40 billion yuan, which contributes to more than 1.60% of the national health expenditure[34]. ASCVD has become a major public health problem worldwide, making the prevention and treatment of CVD urgent.

For peer review only - http://bmjopen.bmj.com/site/about/guidelines.xhtml

A large amount of evidence shows that the pathogenesis of AS is mainly related to the adaptive immune-inflammatory response. AS contains IgG antibodies against LDL, oxidized LDL (ox-LDL) and apolipoprotein B (ApoB)[35]. Some studies suggest that IgG antibody titer is positively correlated with AS, whether in mice or humans[36], and that anti-ApoB IgG antibody can aggravate AS[37]. *In vitro* experiments demonstrate that IgG and very low-density lipoprotein (VLDL) are responsible for forming arterial lesions[38]. As an immunoinflammatory biomarker, IgG glycosylation is effective in differentiating patients with dyslipidemia from the healthy, displaying an area under the curve (AUC) of approximately 0.7 in a cross-sectional study of Chinese Han[25]. Removal of glycans contributes to the disappearance of the anti-inflammatory properties of IgG[39]. While the enrichment of sialic acid is responsible for a tenfold increase in the anti-inflammatory properties of IgG[40]. Therefore, control of the adaptive immune response through immune regulatory strategies or vaccination is a potential therapeutic direction for AS.

Glycoprotein acetylation (GlycA), a more reliable compound biological marker of systemic inflammation, has potential value in predicting CVD outcomes and reflecting inflammation[41 42 43 44 26]. However, GlycA is a glycan signal of a single measure based on nuclear magnetic resonance (NMR). Therefore, the association between IgG N-glycosylation with richer glycoprotein traits and CVD composite events will be assessed in this study. If the results of this study show a significant correlation between IgG glycosylated traits and actual clinical CVD composite events,

For peer review only - http://bmjopen.bmj.com/site/about/guidelines.xhtml

it will provide further evidence on the role of glycosylation modification and immune-inflammatory response in AS, as well as be a potential biomarker better than GlycA in predicting CVD events in the clinic. In addition, clarifying the correlation between IgG and AS, and revealing the mechanism of atherogenesis are essential for providing new directions for the treatment and prevention of CVD. Our study will provide important scientific significance and socio-economic value for improving the quality of life, decreasing the mortality rate and the socio-economic burden of CVD patients.

However, some limitations in this study exist. Firstly, the participants are recruited at a single center and represent only one ethnic group, Han Chinese, in the Chaoshan area of Guangdong Province, and thus lack ethnic diversity. Further, a multi-center cohort study is required for application of results to the general population. Secondly, follow-up bias is inevitable in this prospective cohort study due to the existence of subjects who may migrate, leave, or withdraw from this cohort, although the authors will try their best to control it.

FULL REFERENCES

1. Wang Y, Xie Y, Zhang A, et al. Exosomes: An emerging factor in atherosclerosis. *Biomed Pharmacother* 2019;115:108951. doi: 10.1016/j.biopha.2019.108951 [published Online First: 2019/05/12]
2. Libby P, Buring JE, Badimon L, et al. Atherosclerosis. *Nat Rev Dis Primers* 2019;5(1):56. doi: 10.1038/s41572-019-0106-z [published Online First: 2019/08/20]
3. Gupta M, Blumenthal C, Chatterjee S, et al. Novel emerging therapies in atherosclerosis targeting lipid metabolism. *Expert Opin Investig Drugs* 2020;29(6):611-22. doi: 10.1080/13543784.2020.1764937 [published Online First: 2020/05/05]
4. Kattoor AJ, Pothineni NVK, Palagiri D, et al. Oxidative Stress in Atherosclerosis. *Current atherosclerosis reports* 2017;19(11):42. doi: 10.1007/s11883-017-0678-6 [published Online

For peer review only - http://bmjopen.bmj.com/site/about/guidelines.xhtml

First: 2017/09/19]
5. Wang Y, Ge S, Yan Y, et al. China suboptimal health cohort study: rationale, design and baseline characteristics. *J Transl Med* 2016;14(1):291. doi: 10.1186/s12967-016-1046-y [published Online First: 2016/10/16]
6. Cao W, Zheng D, Zhang J, et al. Association between telomere length in peripheral blood leukocytes and risk of ischemic stroke in a Han Chinese population: a linear and non-linear Mendelian randomization analysis. *J Transl Med* 2020;18(1):385. doi: 10.1186/s12967-020-02551-1 [published Online First: 2020/10/14]
7. Sage AP, Mallat Z. Readapting the adaptive immune response - therapeutic strategies for atherosclerosis. *Br J Pharmacol* 2017;174(22):3926-39. doi: 10.1111/bph.13700 [published Online First: 2017/01/05]
8. Libby P, Lichtman AH, Hansson GK. Immune effector mechanisms implicated in atherosclerosis: from mice to humans. *Immunity* 2013;38(6):1092-104. doi: 10.1016/j.immuni.2013.06.009 [published Online First: 2013/07/03]
9. Ridker PM, Everett BM, Thuren T, et al. Antiinflammatory Therapy with Canakinumab for Atherosclerotic Disease. *The New England journal of medicine* 2017;377(12):1119-31. doi: 10.1056/NEJMoa1707914 [published Online First: 2017/08/29]
10. Centa M, Jin H, Hofste L, et al. Germinal Center-Derived Antibodies Promote Atherosclerosis Plaque Size and Stability. *Circulation* 2019;139(21):2466-82. doi: 10.1161/circulationaha.118.038534 [published Online First: 2019/03/22]
11. Molinari M. N-glycan structure dictates extension of protein folding or onset of disposal. *Nat Chem Biol* 2007;3(6):313-20. doi: 10.1038/nchembio880 [published Online First: 2007/05/19]
12. Ohtsubo K, Marth JD. Glycosylation in cellular mechanisms of health and disease. *Cell* 2006;126(5):855-67. doi: 10.1016/j.cell.2006.08.019 [published Online First: 2006/09/09]
13. Parekh RB, Dwek RA, Sutton BJ, et al. Association of rheumatoid arthritis and primary osteoarthritis with changes in the glycosylation pattern of total serum IgG. *Nature* 1985;316(6027):452-7. doi: 10.1038/316452a0 [published Online First: 1985/08/01]
14. Yi CH, Weng HL, Zhou FG, et al. Elevated core-fucosylated IgG is a new marker for hepatitis B virus-related hepatocellular carcinoma. *Oncoimmunology* 2015;4(12):e1011503. doi: 10.1080/2162402x.2015.1011503 [published Online First: 2015/11/21]
15. Bones J, Byrne JC, O'Donoghue N, et al. Glycomic and glycoproteomic analysis of serum from patients with stomach cancer reveals potential markers arising from host defense response mechanisms. *J Proteome Res* 2011;10(3):1246-65. doi: 10.1021/pr101036b [published Online First: 2010/12/15]
16. Qian Y, Wang Y, Zhang X, et al. Quantitative analysis of serum IgG galactosylation assists differential diagnosis of ovarian cancer. *J Proteome Res* 2013;12(9):4046-55. doi: 10.1021/pr4003992 [published Online First: 2013/07/17]
17. Costa J, Streich L, Pinto S, et al. Exploring Cerebrospinal Fluid IgG N-Glycosylation as Potential Biomarker for Amyotrophic Lateral Sclerosis. *Mol Neurobiol* 2019;56(8):5729-39. doi: 10.1007/s12035-019-1482-9 [published Online First: 2019/01/24]
18. Giron LB, Azzoni L, Yin X, et al. Hepatitis C virus modulates IgG glycosylation in HIV co-infected antiretroviral therapy suppressed individuals. *Aids* 2020;34(10):1461-66. doi: 10.1097/qad.0000000000002558 [published Online First: 2020/07/18]
19. Šimurina M, de Haan N, Vučković F, et al. Glycosylation of Immunoglobulin G Associates With

For peer review only

Clinical Features of Inflammatory Bowel Diseases. *Gastroenterology* 2018;154(5):1320-33.e10. doi: 10.1053/j.gastro.2018.01.002 [published Online First: 2018/01/09]

20. Lauc G, Huffman JE, Pučić M, et al. Loci associated with N-glycosylation of human immunoglobulin G show pleiotropy with autoimmune diseases and haematological cancers. *PLoS Genet* 2013;9(1):e1003225. doi: 10.1371/journal.pgen.1003225 [published Online First: 2013/02/06]
21. Maverakis E, Kim K, Shimoda M, et al. Glycans in the immune system and The Altered Glycan Theory of Autoimmunity: a critical review. *J Autoimmun* 2015;57:1-13. doi: 10.1016/j.jaut.2014.12.002 [published Online First: 2015/01/13]
22. Chan AC, Carter PJ. Therapeutic antibodies for autoimmunity and inflammation. *Nat Rev Immunol* 2010;10(5):301-16. doi: 10.1038/nri2761 [published Online First: 2010/04/24]
23. Washburn N, Schwab I, Ortiz D, et al. Controlled tetra-Fc sialylation of IVIg results in a drug candidate with consistent enhanced anti-inflammatory activity. *Proc Natl Acad Sci U S A* 2015;112(11):E1297-306. doi: 10.1073/pnas.1422481112 [published Online First: 2015/03/04]
24. Nimmerjahn F, Anthony RM, Ravetch JV. Agalactosylated IgG antibodies depend on cellular Fc receptors for in vivo activity. *Proc Natl Acad Sci U S A* 2007;104(20):8433-7. doi: 10.1073/pnas.0702936104 [published Online First: 2007/05/09]
25. Liu D, Chu X, Wang H, et al. The changes of immunoglobulin G N-glycosylation in blood lipids and dyslipidaemia. *J Transl Med* 2018;16(1):235. doi: 10.1186/s12967-018-1616-2 [published Online First: 2018/08/31]
26. Menni C, Gudelj I, Macdonald-Dunlop E, et al. Glycosylation Profile of Immunoglobulin G Is Cross-Sectionally Associated With Cardiovascular Disease Risk Score and Subclinical Atherosclerosis in Two Independent Cohorts. *Circulation research* 2018;122(11):1555-64. doi: 10.1161/circresaha.117.312174 [published Online First: 2018/03/15]
27. Chan AW, Tetzlaff JM, Altman DG, et al. SPIRIT 2013 statement: defining standard protocol items for clinical trials. *Ann Intern Med* 2013;158(3):200-7. doi: 10.7326/0003-4819-158-3-201302050-00583 [published Online First: 2013/01/09]
28. Kotruchin P, Hoshide S, Kario K. Carotid atherosclerosis and the association between nocturnal blood pressure dipping and cardiovascular events. *J Clin Hypertens (Greenwich)* 2018;20(3):450-55. doi: 10.1111/jch.13218 [published Online First: 2018/02/17]
29. Liu D, Zhao Z, Wang A, et al. Ischemic stroke is associated with the pro-inflammatory potential of N-glycosylated immunoglobulin G. *J Neuroinflammation* 2018;15(1):123. doi: 10.1186/s12974-018-1161-1 [published Online First: 2018/04/28]
30. Shi J, Luo D, Weng H, et al. Optimally estimating the sample standard deviation from the five-number summary. *Res Synth Methods* 2020;11(5):641-54. doi: 10.1002/jrsm.1429 [published Online First: 2020/06/21]
31. Luo D, Wan X, Liu J, et al. Optimally estimating the sample mean from the sample size, median, mid-range, and/or mid-quartile range. *Stat Methods Med Res* 2018;27(6):1785-805. doi: 10.1177/0962280216669183 [published Online First: 2016/09/30]
32. Wan X, Wang W, Liu J, et al. Estimating the sample mean and standard deviation from the sample size, median, range and/or interquartile range. *BMC Med Res Methodol* 2014;14:135. doi: 10.1186/1471-2288-14-135 [published Online First: 2014/12/20]
33. Liu D, Xu X, Li Y, et al. Immunoglobulin G N-Glycan Analysis by Ultra-Performance Liquid

For peer review only

Chromatography. *J Vis Exp* 2020(155) doi: 10.3791/60104 [published Online First: 2020/02/06]

34. Hu SS, Kong LZ, Gao RL, et al. Outline of the report on cardiovascular disease in China, 2010. *Biomed Environ Sci* 2012;25(3):251-6. doi: 10.3967/0895-3988.2012.03.001 [published Online First: 2012/07/31]

35. Kimura T, Tse K, Sette A, et al. Vaccination to modulate atherosclerosis. *Autoimmunity* 2015;48(3):152-60. doi: 10.3109/08916934.2014.1003641 [published Online First: 2015/02/17]

36. Björkbacka H, Alm R, Persson M, et al. Low Levels of Apolipoprotein B-100 Autoantibodies Are Associated With Increased Risk of Coronary Events. *Arteriosclerosis, thrombosis, and vascular biology* 2016;36(4):765-71. doi: 10.1161/atvbaha.115.306938 [published Online First: 2016/02/27]

37. Tay C, Liu YH, Kanellakis P, et al. Follicular B Cells Promote Atherosclerosis via T Cell-Mediated Differentiation Into Plasma Cells and Secreting Pathogenic Immunoglobulin G. *Arteriosclerosis, thrombosis, and vascular biology* 2018;38(5):e71-e84. doi: 10.1161/atvbaha.117.310678 [published Online First: 2018/03/31]

38. Sarphie TG. Interactions of IgG and beta-VLDL with aortic valve endothelium from hypercholesterolemic rabbits. *Atherosclerosis* 1987;68(3):199-212. doi: 10.1016/0021-9150(87)90199-7 [published Online First: 1987/12/01]

39. Kaneko Y, Nimmerjahn F, Ravetch JV. Anti-inflammatory activity of immunoglobulin G resulting from Fc sialylation. *Science (New York, NY)* 2006;313(5787):670-3. doi: 10.1126/science.1129594 [published Online First: 2006/08/05]

40. Schwab I, Nimmerjahn F. Intravenous immunoglobulin therapy: how does IgG modulate the immune system? *Nat Rev Immunol* 2013;13(3):176-89. doi: 10.1038/nri3401 [published Online First: 2013/02/16]

41. Duprez DA, Otvos J, Sanchez OA, et al. Comparison of the Predictive Value of GlycA and Other Biomarkers of Inflammation for Total Death, Incident Cardiovascular Events, Noncardiovascular and Noncancer Inflammatory-Related Events, and Total Cancer Events. *Clin Chem* 2016;62(7):1020-31. doi: 10.1373/clinchem.2016.255828 [published Online First: 2016/05/14]

42. Akinkuolie AO, Buring JE, Ridker PM, et al. A novel protein glycan biomarker and future cardiovascular disease events. *Journal of the American Heart Association* 2014;3(5):e001221. doi: 10.1161/jaha.114.001221 [published Online First: 2014/09/25]

43. Lawler PR, Akinkuolie AO, Chandler PD, et al. Circulating N-Linked Glycoprotein Acetyls and Longitudinal Mortality Risk. *Circulation research* 2016;118(7):1106-15. doi: 10.1161/circresaha.115.308078 [published Online First: 2016/03/10]

44. McGarrah RW, Kelly JP, Craig DM, et al. A Novel Protein Glycan-Derived Inflammation Biomarker Independently Predicts Cardiovascular Disease and Modifies the Association of HDL Subclasses with Mortality. *Clin Chem* 2017;63(1):288-96. doi: 10.1373/clinchem.2016.261636 [published Online First: 2016/11/05]

For peer review only

For peer review only - http://bmjopen.bmj.com/site/about/guidelines.xhtml

For peer review only

Trial status Participant recruitment began on 15 July 2021 and will be finished on 31 December 2021.

Acknowledgments We would like to thank Dr. Stanley Li Lin, who is native English speaker from America as a foreign teacher at the Department of Cell Biology and Genetics, SUMC, for his helpful comments and English language editing. We are grateful to Xingyu Wang for his helpful advice of the protocol.

Authors' Contributions CT was responsible for the design and draft of the manuscript. HZ, working as a sonographer, contributed to the collection of clinical information of the participants. GM reviewed this manuscript. XT, acting as a principal investigator, helped to conceive the protocol. The final version of the manuscript was critically reviewed and approved by all authors.

Funding statement This work is supported by the National Natural Science Foundation of China (No. 82073659) and the Provincial Science and Technology Special Fund of Guangdong in 2021(No. 2021123071-1).

Competing interests statement The authors declare that they have no competing interests.

Participants and public involvement Participants and/or the public were not involved in the design, conduct, reporting or dissemination plan of this research.

Participant consent for publication Not required.

Provenance and peer review Not commissioned; externally peer-reviewed.

Open access This is an open access article distributed in accordance with the Creative Commons Attribution Non-Commercial (CC BY- NC 4.0) license, which permits others to distribute, remix, adapt, build upon this work non-commercially, and license their derivative works on different

terms, provided the original work is properly cited, appropriate credit is given, any changes made indicated, and the use is non-commercial. See: http://creativecommons. org/licenses/ by-nc/4.0/.

Consent for publication All authors and investigators gave their consent to publication.

Availability of data and materials The owner of this protocol has access to the final study dataset. The study data will be made public in the EDC system, where participants' names, phone numbers and other personal information will be removed to protect their privacy. Moreover, manuscript(s) will be published in a peer review clinical journal regardless of whether the study results are positive, negative or inconclusive. Upon reasonable request, the public will be allowed to view the data with the permission of the authors or corresponding author.

Word count 3246 words.

Figure 1. Participant distribution in this study.

Figure 2. Schedule of enrollment, distribution and assessments for the study.

For peer review only

For peer review only - http://bmjopen.bmj.com/site/about/guidelines.xhtml

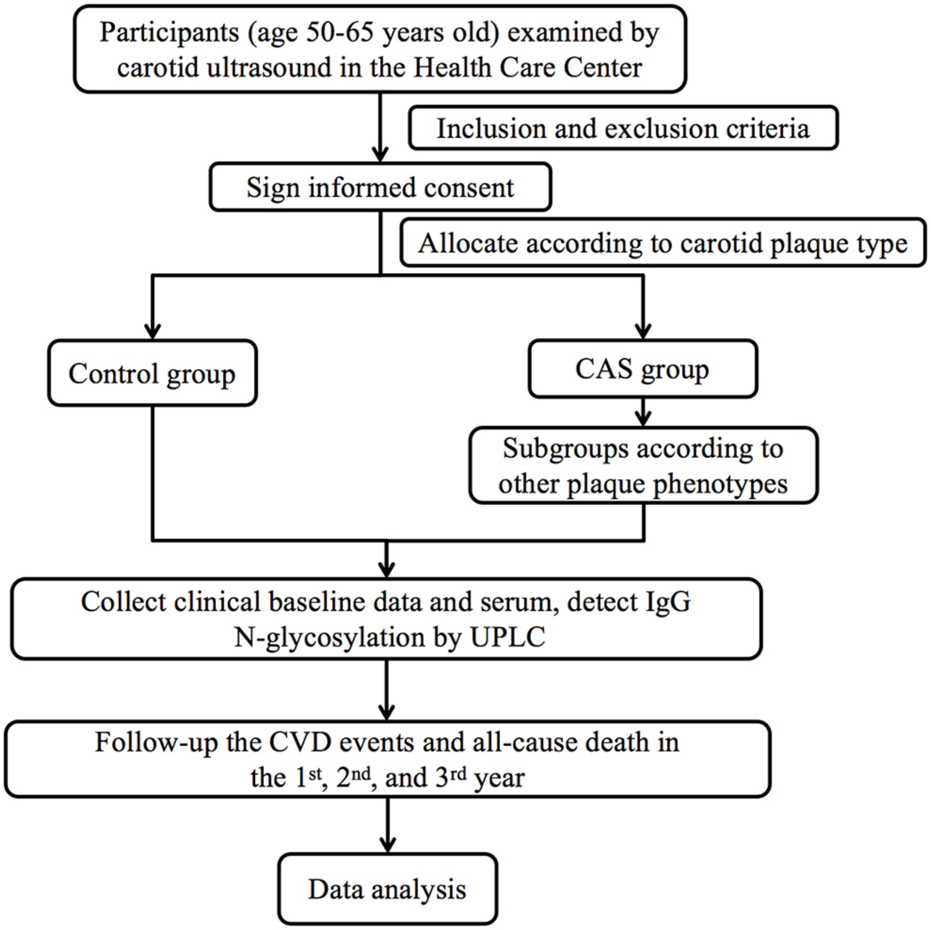

Figure 1. Participant distribution in this study.

375x375mm (72 x 72 DPI)

Study period							
	Enrollment	Distribution	UPLC	Follow-up			
Timepoint	D0	D1	W1	Y1	Y2	Y3	Close-out
Enrollment							
Prescreening	X						
Eligibility screen	X						
Informed consent	X						
Distribution							
Control group		X					
CAS group		X					
Assessments							
Gender		X					
Age		X					
Smoking		X					
Diabetes		X					
Hypotensor		X					
LDL		X					
HDL		X					
TC		X					
TG		X					
Carotid ultrasound							
Size		X					
Number		X					
Situation		X					
Blood flow		X					
Echo		X					
Shape		X					
Stability		X					
Serum		X					
UPLC							
Ig G glycosylation			X	X	X	X	
Outcome							
Nonfatal MI				X	X	X	
CHD death				X	X	X	
Stroke				X	X	X	
All-cause death				X	X	X	
Analysis							X

Figure 2. Schedule of enrollment, distribution and assessments for the study.

90x90mm (300 x 300 DPI)

SPIRIT

STANDARD PROTOCOL ITEMS: RECOMMENDATIONS FOR INTERVENTIONAL TRIALS

SPIRIT 2013 Checklist: Recommended items to address in a clinical trial protocol and related documents*

Section/item	Item No	Description
**Administrative information**		
Title	1	Descriptive title identifying the study design, population, interventions, and, if applicable, trial acronym
Trial registration	2a	Trial identifier and registry name. If not yet registered, name of intended registry
	2b	All items from the World Health Organization Trial Registration Data Set
Protocol version	3	Date and version identifier
Funding	4	Sources and types of financial, material, and other support
Roles and responsibilities	5a	Names, affiliations, and roles of protocol contributors
	5b	Name and contact information for the trial sponsor
	5c	Role of study sponsor and funders, if any, in study design; collection, management, analysis, and interpretation of data; writing of the report; and the decision to submit the report for publication, including whether they will have ultimate authority over any of these activities
	5d	Composition, roles, and responsibilities of the coordinating centre, steering committee, endpoint adjudication committee, data management team, and other individuals or groups overseeing the trial, if applicable (see Item 21a for data monitoring committee)
**Introduction**		
Background and rationale	6a	Description of research question and justification for undertaking the trial, including summary of relevant studies (published and unpublished) examining benefits and harms for each intervention
	6b	Explanation for choice of comparators
Objectives	7	Specific objectives or hypotheses
Trial design	8	Description of trial design including type of trial (eg, parallel group, crossover, factorial, single group), allocation ratio, and framework (eg, superiority, equivalence, noninferiority, exploratory)

For peer review only

For peer review only - http://bmjopen.bmj.com/site/about/guidelines.xhtml

Methods: Participants, interventions, and outcomes

Study setting	9	Description of study settings (eg, community clinic, academic hospital) and list of countries where data will be collected. Reference to where list of study sites can be obtained
Eligibility criteria	10	Inclusion and exclusion criteria for participants. If applicable, eligibility criteria for study centres and individuals who will perform the interventions (eg, surgeons, psychotherapists)
Interventions	11a	Interventions for each group with sufficient detail to allow replication, including how and when they will be administered
	11b	Criteria for discontinuing or modifying allocated interventions for a given trial participant (eg, drug dose change in response to harms, participant request, or improving/worsening disease)
	11c	Strategies to improve adherence to intervention protocols, and any procedures for monitoring adherence (eg, drug tablet return, laboratory tests)
	11d	Relevant concomitant care and interventions that are permitted or prohibited during the trial
Outcomes	12	Primary, secondary, and other outcomes, including the specific measurement variable (eg, systolic blood pressure), analysis metric (eg, change from baseline, final value, time to event), method of aggregation (eg, median, proportion), and time point for each outcome. Explanation of the clinical relevance of chosen efficacy and harm outcomes is strongly recommended
Participant timeline	13	Time schedule of enrolment, interventions (including any run-ins and washouts), assessments, and visits for participants. A schematic diagram is highly recommended (see Figure)
Sample size	14	Estimated number of participants needed to achieve study objectives and how it was determined, including clinical and statistical assumptions supporting any sample size calculations
Recruitment	15	Strategies for achieving adequate participant enrolment to reach target sample size

Methods: Assignment of interventions (for controlled trials)

Allocation:

Sequence generation	16a	Method of generating the allocation sequence (eg, computer-generated random numbers), and list of any factors for stratification. To reduce predictability of a random sequence, details of any planned restriction (eg, blocking) should be provided in a separate document that is unavailable to those who enrol participants or assign interventions

Allocation concealment mechanism	16b	Mechanism of implementing the allocation sequence (eg, central telephone; sequentially numbered, opaque, sealed envelopes), describing any steps to conceal the sequence until interventions are assigned
Implementation	16c	Who will generate the allocation sequence, who will enrol participants, and who will assign participants to interventions
Blinding (masking)	17a	Who will be blinded after assignment to interventions (eg, trial participants, care providers, outcome assessors, data analysts), and how
	17b	If blinded, circumstances under which unblinding is permissible, and procedure for revealing a participant's allocated intervention during the trial
**Methods: Data collection, management, and analysis**		
Data collection methods	18a	Plans for assessment and collection of outcome, baseline, and other trial data, including any related processes to promote data quality (eg, duplicate measurements, training of assessors) and a description of study instruments (eg, questionnaires, laboratory tests) along with their reliability and validity, if known. Reference to where data collection forms can be found, if not in the protocol
	18b	Plans to promote participant retention and complete follow-up, including list of any outcome data to be collected for participants who discontinue or deviate from intervention protocols
Data management	19	Plans for data entry, coding, security, and storage, including any related processes to promote data quality (eg, double data entry; range checks for data values). Reference to where details of data management procedures can be found, if not in the protocol
Statistical methods	20a	Statistical methods for analysing primary and secondary outcomes. Reference to where other details of the statistical analysis plan can be found, if not in the protocol
	20b	Methods for any additional analyses (eg, subgroup and adjusted analyses)
	20c	Definition of analysis population relating to protocol non-adherence (eg, as randomised analysis), and any statistical methods to handle missing data (eg, multiple imputation)
**Methods: Monitoring**		
Data monitoring	21a	Composition of data monitoring committee (DMC); summary of its role and reporting structure; statement of whether it is independent from the sponsor and competing interests; and reference to where further details about its charter can be found, if not in the protocol. Alternatively, an explanation of why a DMC is not needed

For peer review only - http://bmjopen.bmj.com/site/about/guidelines.xhtml

	21b	Description of any interim analyses and stopping guidelines, including who will have access to these interim results and make the final decision to terminate the trial
Harms	22	Plans for collecting, assessing, reporting, and managing solicited and spontaneously reported adverse events and other unintended effects of trial interventions or trial conduct
Auditing	23	Frequency and procedures for auditing trial conduct, if any, and whether the process will be independent from investigators and the sponsor
**Ethics and dissemination**		
Research ethics approval	24	Plans for seeking research ethics committee/institutional review board (REC/IRB) approval
Protocol amendments	25	Plans for communicating important protocol modifications (eg, changes to eligibility criteria, outcomes, analyses) to relevant parties (eg, investigators, REC/IRBs, trial participants, trial registries, journals, regulators)
Consent or assent	26a	Who will obtain informed consent or assent from potential trial participants or authorised surrogates, and how (see Item 32)
	26b	Additional consent provisions for collection and use of participant data and biological specimens in ancillary studies, if applicable
Confidentiality	27	How personal information about potential and enrolled participants will be collected, shared, and maintained in order to protect confidentiality before, during, and after the trial
Declaration of interests	28	Financial and other competing interests for principal investigators for the overall trial and each study site
Access to data	29	Statement of who will have access to the final trial dataset, and disclosure of contractual agreements that limit such access for investigators
Ancillary and post-trial care	30	Provisions, if any, for ancillary and post-trial care, and for compensation to those who suffer harm from trial participation
Dissemination policy	31a	Plans for investigators and sponsor to communicate trial results to participants, healthcare professionals, the public, and other relevant groups (eg, via publication, reporting in results databases, or other data sharing arrangements), including any publication restrictions
	31b	Authorship eligibility guidelines and any intended use of professional writers
	31c	Plans, if any, for granting public access to the full protocol, participant-level dataset, and statistical code

For peer review only

Appendices

Informed consent materials	32	Model consent form and other related documentation given to participants and authorised surrogates
Biological specimens	33	Plans for collection, laboratory evaluation, and storage of biological specimens for genetic or molecular analysis in the current trial and for future use in ancillary studies, if applicable

*It is strongly recommended that this checklist be read in conjunction with the SPIRIT 2013 Explanation & Elaboration for important clarification on the items. Amendments to the protocol should be tracked and dated. The SPIRIT checklist is copyrighted by the SPIRIT Group under the Creative Commons "Attribution-NonCommercial-NoDerivs 3.0 Unported" license.

For peer review only
